# Serum follicle-stimulating hormone level is associated with human epidermal growth factor receptor type 2 and Ki67 expression in post-menopausal females with breast cancer

**DOI:** 10.3892/ol.2013.1516

**Published:** 2013-08-07

**Authors:** JUN ZHOU, YIDING CHEN, YITING HUANG, JINPEI LONG, FANG WAN, SUZHAN ZHANG

**Affiliations:** 1 Key Laboratory of Cancer Prevention and Intervention, China National Ministry of Education, Key Laboratory of Molecular Biology in Medical Sciences of Zhejiang, Cancer Institute of Zhejiang University, Hangzhou, Zhejiang 310009, P.R. China; 2 Department of Surgery, The Women's Hospital of Zhejiang University, Hangzhou, Zhejiang 310006, P.R. China; 3 Department of Pathology and Pathophysiology, School of Zhejiang University, Hangzhou 310012, P.R. China

**Keywords:** breast cancer, human epidermal growth factor receptor 2, Ki67, follicle-stimulating hormone, post-menopausal

## Abstract

The present study aimed to determine the association between levels of the gender hormones, follicle-stimulating hormone (FSH), luteinizing hormone (LH), progesterone (P) and prolactin (PRL), and two breast cancer molecular markers, human epidermal growth factor receptor 2 (Her-2) and Ki67, in post-menopausal patients with breast cancer. A retrospective study of the serum hormone levels of FSH, LH, P and PRL and the expression status of Her-2 and Ki67 was performed using 187 post-menopausal females with breast cancer. Her-2^+^ breast cancer patients exhibited higher serum FSH levels compared with Her-2^−^ patients (69.47±3.219 vs. 58.56±1.516 IU/l). The patients with high Ki67 expression [immunohistochemistry (IHC), 3+] displayed higher FSH (72.51±4.616 vs. 60.53±1.476 IU/l) and LH (32.33±1.916 vs. 26.98±0.8852 IU/l) levels than those with lower Ki67 expression. No correlation was identified between the FSH, LH, P and PRL hormone levels, tumor stages and lymphovascular invasion (LVI). In conclusion, a higher serum FSH level was identified in Her-2^+^ post-menopausal patients with breast cancer. Higher serum FSH and LH levels were also observed in breast cancer patients with high Ki67 expression. FSH and LH may function in the progression of breast cancer.

## Introduction

Breast cancer is one of the most common cancers in females and one of the main causes of cancer-related mortality worldwide (1). In China, the incidence and mortality rates of breast cancer have continuously increased (2,3). Chinese patients with breast cancer exhibit a more invasive ductal carcinoma with larger tumor sizes and higher human epidermal growth factor receptor 2 (Her-2) overexpression than patients from the West (4).

Her-2 regulates cell growth, survival and differentiation via interlinked signal transduction. Her-2 amplification and overexpression have been reported in 15–30% of all breast cancer cases and are associated with a poorer prognosis and more aggressive clinical manifestations (5,6). The exact mechanism of Her-2 overexpression remains unclear.

The nuclear-associated antigen Ki67 protein may be detected in the active phases of the cell cycle in late G_1_, S, G_2_ and M phases, but not in the resting phase (7). The overexpression of Ki67 corresponds to the high proliferation rate of tumor cells. Ki67 is used as the main marker to distinguish between luminal A (Ki67 <14%) and luminal B (Ki67 ≥14%) breast cancers. Luminal B indicates that the tumor is more aggressive and requires chemotherapy (8).

Studies have identified that hormones other than estradiol (E2) may be associated with an increased risk of breast cancer. High serum prolactin (PRL) levels have been reported in pre-menopausal females with breast cancer (9) and circulating PRL levels are positively correlated with the risk of breast cancer (10). Although gene scans have shown that the expression of luteinizing hormone/choriogonadotropin receptor (LH/CGR) in breast cancer is either undetectable or very low (11), studies have identified that LHCGR may be detected in breast cancer cells (12–14) and that LH participates in the tumor progression of breast cancer using LHCGR. Nearly 40% of patients with breast cancer exhibit an increased human chorionic gonadotropin (hCG)-immunoreactivity in the serum (15,16). However, few studies have focused on the association between follicle-stimulating hormone (FSH) and breast cancer.

FSH stimulates follicle growth and development in the ovaries and produces the maximum amount of mature spermatozoa in the testes. FSH and its corresponding receptor (FSHR) have an important function in various cancers, including prostate (17), endometrial (18) and ovarian (19) cancer. FSH-FSHR induces cancer cell proliferation, differentiation and metastasis by activating adenylyl cyclase, thereby resulting in increased cAMP levels (20,21). The overexpression of FSHR may be associated with Her-2 overexpression in ovarian cancer (22). Although FSHR expression has not been identified in primary tissues of breast cancer (23), high FSH levels have been associated with a significantly poor prognosis in patients with premenopausal breast cancer (24). FSH has also been linked to breast cancer cell proliferation and an increased risk of breast cancer development in females who have undergone infertility treatments (25). However, few studies have focused on the association between the serum level of FSH and the expression status of Her-2 and Ki67.

The present study hypothesized that gonadotropic hormone has a function in the proliferation of breast cancer cells. The association between serum hormonal levels and the expression status of two relative breast cancer proliferation molecular markers, Her-2 and Ki67, was retrospectively analyzed in 187 post-menopausal females with breast cancer.

## Materials and methods

### Patients

The data of 187 post-menopausal breast cancer patients were collected from The Women's Hospital of Zhejiang University (Zhejiang, China) between January 2007 and October 2012. The post-menopausal standard was based on the National Comprehensive Cancer Network Guidelines of 2012. The serum hormonal levels of FSH, LH, progesterone (P) and PRL were evaluated at the initial admission of the patients. The patients who were administered chemotherapy, radiotherapy or hormonal replacement therapy prior to the surgery were excluded, as the therapies may have affected their hormonal levels. The study was approved by The Women's Hospital of Zhejiang University Ethics Committee. Written informed consent was obtained from the patients.

### Determination of circulating levels of FSH, LH, P and PRL

Venous blood was collected in 6-ml ethylenediaminetetraacetic acid (EDTA) tubes at 6 a.m. and analyzed within 24 h. The circulating hormone levels, including those of FSH, LH, P and PRL, were measured using enzyme immunoassays (Roche Diagnostics, Mannheim, Germany) on an E170 module.

### Evaluation of Her-2 and Ki67 by immunohistochemistry (IHC)

The tumor grades were assessed using the tumor-node-metastasis (TNM) staging system. The slides were re-examined by two expert pathologists to confirm the tumor type, size and grade and the presence of lymphovascular invasion (LVI). The classification of Ki67 was determined based on two methods. The first method divided patients into two groups based on the positive rates of <14% (IHC, 0 or 1+) or ≥14% (IHC, 2+ or 3+). The second method divided patients based on the IHC results of 3+ as group 1 and 2+/1+/− as group 2.

### Statistical analysis

The statistical analysis was performed using SPSS 19.0 software (SPSS, Inc., Chicago, IL, USA). A Mann-Whitney U test was performed to determine the association between the hormonal levels and the expression rates of Her-2 and Ki67. A one-way ANOVA was performed to evaluate the association between the hormonal level, LVI and the tumor stage. All of the reported P-values were two-sided. P<0.05 was considered to indicate a statistically significant difference.

## Results

The clinical and pathological features of the 187 post-menopausal patients with breast cancer are summarized in Table I. All 187 patients underwent breast cancer surgery without previous chemotherapy, radiotherapy or any other hormonal replacement therapy. All patients were pathologically diagnosed with breast cancer.

**Figure 1:**
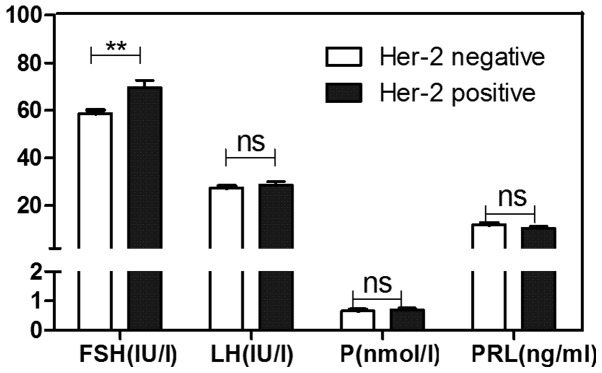
Circulating levels of hormones in Her-2^+^ and Her-2^−^ post-menopausal females with breast cancer. Data are presented as the mean ± SEM; n=56 and n=131 as positive and negative, respectively; ^**^P<0.01. FSH, follicle-stimulating hormone; LH, luteinizing hormone; P, progesterone; PRL, prolactin; Her-2, human epidermal growth factor receptor type 2; ns, not significant.

The FSH level was strongly associated with the Her-2 status (P=0.004; Fig. 1). The Her-2^+^ patients exhibited higher FSH levels than the Her-2^−^ patients (69.47±3.219 vs. 58.56±1.516 IU/l). In contrast, the LH, P and PRL hormone levels were not exhibited with significant differences between the Her-2^+^ and Her-2^−^ patients with post-menopausal breast cancer.

**Figure 2:**
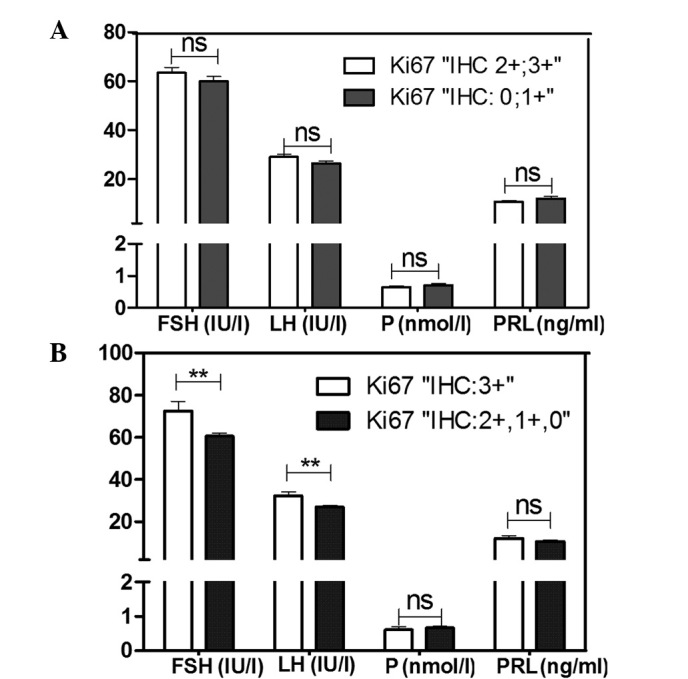
Circulating levels of hormones grouped by Ki67 levels in post-menopausal females with breast cancer. (A) Levels are grouped according to the IHC results, 0; 1^+^ and 2^+^; 3^+^ (n=89 and 98; ^**^P<0.01). (B) Levels are grouped according to the IHC results, 0 and 1^+^; 2^+^ and 3^+^ (n=150 and n=37, repectively; ^**^P<0.01). Data are presented as mean ± SEM. IHC, immunohistochemistry; FSH, follicle-stimulating hormone; LH, luteinizing hormone; P, progesterone; PRL, prolactin; ns, not significant.

A cut-off point of 14% was selected for Ki67. The results revealed that FSH, LH, P and PRL were not significantly different between the two groups (Fig. 2A). In contrast, group 1 exhibited higher FSH (72.51±4.616 vs. 60.53±1.476) and LH (32.33±1.916 vs. 26.98±0.885) levels than group 2 (Fig. 2B).

The FSH, LH, P and PRL levels did not change significantly in tumor stage groups I–IV compared with stage 0 (Table II). The serum levels of the hormones were evaluated between the various LVI stage groups, however, no significant difference was identified in the circulating FSH, LH, P and PRL levels in the high LVI groups compared with the LVI-negative group.

## Discussion

The effect of hormones on tumorigenesis and tumor progression, particularly the function of the estrogen signal pathway in breast cancer, has been widely discussed. However, the pathogenesis and progression of breast cancer remains unclear. Furthermore, the specific functions of other hormones, including FSH and LH, have not been fully elucidated with regard to the progression of breast cancer. The present study analyzed 187 post-menopausal breast cancer patients to determine whether or not the serum hormonal levels of FSH, LH, P and PRL were associated with the expression of two key molecular markers, Ki67 and Her-2. Premenopausal patients were excluded as their hormonal levels are affected by their physiological cycle and thus their basic hormonal level is difficult to determine in the surgery department of the hospital. The serum FSH levels differed between the Her-2^+^ and Her-2^−^ patients, and higher FSH levels were identified in the Her-2^+^ patients. A higher serum FSH level was also identified in patients with high Ki67 expression (IHC, 3+). The serum LH level exhibited no significant difference based on Her-2 expression, but a higher serum LH level was observed in the patients with a high Ki67 (IHC, 3+).

FSH and LH belong to a family of glycoprotein hormones, which also include placental hCG and thyroid-stimulating hormone (TSH). FSH and LH are key regulators of reproductive function in the endocrine system and regulate steroidogenesis and gametogenesis in the ovary and the testis. FSH stimulates follicular cell activity through FSHR. FSHR expression is restricted to the sterol cells in the testis and the granulose cells in the ovary (26,27). FSHR expression has been identified in cancer cells. However, the exact function of gonadotropin and its molecular mechanism in the formation and development of tumors has not yet been fully characterized.

The FSH and LH receptors belong to the super family of G protein-coupled receptors (GPCRs). However, these hormones are unique as they have a large ectodomains that contain a leucine-rich repeat, which is significant for ligand binding. FSHR expression has been identified in various cancer cells, but rarely in breast cancer tissues or cell lines. However, numerous leucine-rich GPCRs (LGRs) have been identified in the human genome (28). In addition to FSHR, other LGR subgroups are able to transmit signals from gonadotropins (28,29). The expression of leucine-containing GPCRs should be detected in breast cancer as FSH is widely distributed in the cytoplasm of epithelial cells in breast cancer, in which higher levels of FSH are observed in benign mammary tumors and breast cancer compared with normal cells (30). Further studies are required to determine whether or not FSH stimulates Her-2 expression and cell proliferation by the LGR subgroup. FSH may function in the malignant transformation of breast cancer via a specific receptor, but not the traditional FSHR.

Furthermore, the menopause affects Her-2 expression in breast cancer (31). One study focused on FSH and Her-2 in breast cancer (32), while certain studies have revealed that FSH stimulates Her-2 expression via specific signaling pathways, including cAMP, in ovarian cancer (22,33,34). In the present study, patients with high Ki67 expression (IHC, 3+) exhibited higher serum FSH and LH levels. A total of 10 ng/ml FSH was able to upregulate the expression of Her-2 and Ki67 at the transcriptional level in 24 h in a breast cancer cell line *in vitro* (data not shown). Thus, gonadotropins are able to directly or indirectly promote cell proliferation in breast cancer.

To determine whether or not FSH is an independent prognostic marker, the association between FSH or LH and the overall survival (OS) or relapse-free survival (RFS) of the patients was evaluated in the present study. However, the data in this study was acquired from patients who were diagnosed with breast cancer within the past 5 years. Thus, OS/RFS could not be sufficiently evaluated in the study. Future studies with regard to this topic are required.

In conclusion, in the present cases of post-menopausal breast cancer, the Her-2^+^ patients were observed to have a higher serum FSH level than the Her-2^−^ patients. The patients with high Ki67 expression (IHC, 3^+^) exhibited higher serum FSH and LH levels. In addition to E2, FSH and LH may have significant functions in breast cancer progression. Thus, further studies are required to determine the exact mechanism at the molecular level.

## Figures and Tables

**Table I:** Clinical and pathological features of patients (n=187).

Features	Value
Age, years	
Median	62
Range	47–83
FSH, IU/l	
Mean	61.83
Range	8.82–149.6
LH, IU/l	
Mean	27.73
Range	0.1–77.92
P, mmol/l	
Mean	0.673
Range	0.05–2.84
PRL, ng/ml	
Mean	11.38
Range	0.975–61
Her-2, n (%)	
Positive	56 (29.9)
Negative	131 (70.1)
Ki67, n (%)	
Method 1	
<14%	89 (47.6)
≥14%	98 (52.4)
Method 2	
negative	14 (7.5)
1+	75 (40.1)
2+	61 (32.6)
3+	37 (19.8)
LVI, n (%)	
Negative	111 (59.4)
≤3	39 (20.9)
4–9	26 (13.9)
≥10	11 (5.9)
Tumor stage, n (%)	
0	13 (7.0)
I	25 (13.4)
IIa/IIb	76 (40.6)/31 (16.6)
IIIa/IIIb	25 (13.4)/15 (8.0)
IV	2 (1.1)

FSH, follicle-stimulating hormone; LH, luteinizing hormone; P, progesterone; PRL, prolactin; Her-2, human epidermal growth factor receptor type 2; LVI, lymphovascular invasion.

**Table II:** Correlation between the serum hormonal levels of FSH, LH, P and PRL, tumor status and LVI in post-menopausal patients with breast cancer.

	Tumor stage					LVI			
		
Hormone	0	I	IIa/IIb	IIIa/IIIb	IV	0	≤3	4–9	≥10
FSH		0.909	1.000	0.979	0.424		0.976	0.730	0.999
LH		1.000	0.624	0.817	0.839		0.646	0.997	0.994
P		0.377	0.286	0.282	0.530		1.000	0.561	0.988
PRL		0.670	1.000	0.959	0.919		0.995	0.555	0.979

FSH, follicle-stimulating hormone; LH, luteinizing hormone; P, progesterone; PRL, prolactin; LVI, lymphovascular invasion. Data are represented as P-values relative to Stage 0 and LVI stage 0.
